# Adhesion of *Candida albicans* on PTFE membranes used in guided bone regeneration

**DOI:** 10.1002/cre2.902

**Published:** 2024-07-16

**Authors:** Adel Al‐Asfour, Maribasappa Karched, Syed Saad Bin Qasim, Gregor‐Georg Zafiropoulos

**Affiliations:** ^1^ Department of Surgical Sciences, College of Dentistry Kuwait University Safat Kuwait; ^2^ Department of Bioclinical Sciences, College of Dentistry Kuwait University Safat Kuwait

**Keywords:** *C. albicans*, guided bone regeneration/GBR, PTFE membranes

## Abstract

**Objectives:**

Guided bone regeneration (GBR) is a core procedure used to regenerate bone defects. The aim of the study was to investigate the adherence of *Candida albicans* on six commercially available polytetrafluoroethylene (PTFE) membranes used in GBR procedures and the subsequent clinical consequences.

**Materials and Methods:**

Six commercially available PTFE membranes were tested. Two of the membranes had a textured surface and the other four a plane, nontextured one. *C. albicans* (ATCC 24433) was cultured for 24 h, and its cell surface hydrophobicity was assessed using a modified method. *C. albicans* adhesion to membrane discs was studied by scanning electron microscopy (SEM) and real‐time polymerase chain reaction (PCR).

**Results:**

*C. albicans* was found to be hydrophobic (77.25%). SEM analysis showed that *C. albicans* adherence to all membranes examined was characterized by patchy, scattered, and small clustered patterns except for one nontextured membrane with a most rough surface in which a thick biofilm was observed. Real‐time PCR quantification revealed significantly greater adhesion of *C. albicans* cells to PTFE membranes than the control membrane (*p* ≤ .001) with the membranes having a textured surface exhibiting the highest count of 2680 × 10^4^ cells/ml compared to the count of 707 × 10^4^ cells/mL on those with a nontextured one (*p* ≤ .001). One membrane with nontextured surface, but with most rough surface was found to exhibit the highest count of 3010 × 10^4^ cells/ml (*p* ≤ .05).

**Conclusion:**

The results of this study indicate that *C. albicans* adhesion on membranes' surfaces depends on the degree of surface roughness and/or on the presence of a texture. Textured PTFE membranes and/or membranes high roughness showed significantly more adhered *C. albicans* cells. These findings can impact the surgeon's choice of GBR membrane and postoperative maintenance.

## 1 INTRODUCTION

A lack of horizontal and/or vertical bone in implant sites may cause major clinical problems and needs to be corrected before or simultaneously to implant placement (Elgali et al., 2017; Simion et al., 2007). For this purpose, guided bone regeneration (GBR) is performed as a core procedure to regenerate a bone defect (Retzepi & Donos, 2010; Simion et al., 2007). In GBR, nonresorbable polytetrafluoroethylene (PTFE; classified in expand [e‐PTFE] and dense [d‐PTFE]) barrier membranes alone or combined with bone grafts are used to cover a bone defect which has lost its volumetric tissue providing a shielding effect and the desired space for tissue regeneration (Kim & Ku, 2020; Retzepi & Donos, 2010; Vroom et al., 2022). The use of bioresorbable membranes in GBR surgery requires a primary closure over the bone defect. The advantage of the resorbable membranes is that a second surgery for their removal is not necessary. PTFE membranes (especially d‐PTFE ones) could stay partially exposed during a healing period of 3–6 weeks. During this period, oral microorganisms could attach and grow on the membranes' surface. Furthermore, PTFE barriers provide mechanical stability of the graft and wound and create stiffness for space maintenance (Alauddin et al., 2022; Hoffmann et al., 2008; Liu & Kerns, 2014; Rathnayake et al., 2019). For these reasons, we examined only nonresorbable PTFE membranes. Bone regeneration by GBR depends on the migration of pluripotential and osteogenic cells (e.g., osteoblasts from adjacent bone and/or bone marrow) to the bone defect site and exclusion of epithelial cells and fibroblasts. This guided process also entails angiogenesis and osteogenic cell migration, which proceeds from the periphery toward the central part of the bone defect, resulting in the creation of well‐vascularized, granulated tissue upon healing (Elgali et al., 2017; Khojasteh et al., 2017). A common feature of the PTFE membranes is a porosity ranging between <8 and 300 μm, which could facilitate cell attachment and also aid microbial biofilm formation, proliferation, and possible microbial penetration from the oral environment to the bone defect (Lundgren et al., 1998; Selvig et al., 1990).

Among the PTFE membranes, the e‐PTFE have a pore size of 5–30 µm which enables them to be stretched, but they have to be covered during the healing period. On the other hand, d‐PTFE membranes have a dense structure, they do not expand, have low porosity (0.2 μm) and because of that are thought to be more resistant to microbial penetration and could stay partially exposed (Hoffmann et al., 2008; Vroom et al., 2022; Waasdorp & Feldman, 2013).

Less favorable outcomes occur when microbial infections develop after membranes' exposure, requiring, in certain cases, early removal of the barrier. Exposure may permit the communication between the oral environment and newly forming tissues which may increase the potential for infection. Studies have demonstrated that oral microorganisms frequently colonize GBR membranes and that this microbial adherence adversely influences the final clinical result (Lim et al., 2018; Nowzari & Slots, 1994).

The colonization of different d‐PTFE membranes by various bacterial species, such as *Streptococcus mutans*, *Streptococcus oralis*, *Veilonella parvula*, and *Aggregatibacter actinomycetemcomitans*, has been widely reported in the literature (Begic et al., 2022; Grevstad & Leknes, 1993; Sela et al., 1999). The biofilm formation capacity of *Candida albicans* on PTFE in medical devices was investigated by da Rocha et al. (2021) and on the PTFE filling of screw access channels in implant‐supported prostheses by Ramidan et al. (2022). Both studies demonstrated the ability of *C. albicans* to form biofilms on this material. However, to the authors' knowledge, this study is the first to examine the adhesion of *C. albicans* on PTFE membranes used in GBR procedures. For this reason, aim of this in vitro study was to investigate the adhesion of *C. albicans* cells to six commercially available d‐PTFE membranes used in GBR procedures.

## 2 MATERIALS AND METHODS

### 2.1 Membrane specifications

Six commercially available non‐Titanium reinforced PTFE membranes which are regularly used in GBR surgeries were examined. All PTFE membranes were examined only from the soft tissue facing surface. Immobilon®‐P membrane was used as a control membrane (Table 1).

**Table 1:** Specifications of the membranes used.

Membranes	Structure	Surface	Manufacturer
Cytoplast^TM^ TXT‐200	hd‐PTFE	TXT	Osteogenics Biomedical
OsseoGuard®‐TXT	hd‐PTFE	TXT	Zimmer Biomet
permamem®	hd‐PTFE	NTXT	Botiss Biomaterials
Surgitime	PTFE	NTXT	Bionnovation Biomedical
OsseoGuard®‐NTXT	hd‐PTFE	NTXT	Zimmer Biomet
NeoGen®	Dual e‐PTFE	NTXT	Neoss Group
Immobilon®‐P^a^	PVDF	Plain	Merck

Abbreviations: hd, high density; NTXT, nontextured; PTFE, polytetrafluoroethylene; PVDF®‐P, polyvinylidene fluoride; TXT, textured.

^a^ Served as a control.

### 2.2 Candida strain and culture conditions

*Candida albicans* ATCC 24433 strain was cultured on Sabouraud dextrose agar (SDA; BD) aerobically at 37°C for 24 h. The culture was observed under a stereo microscope to confirm the colony morphology and to check for contamination before using it in experiments.

### 2.3 Attachment assay

Under sterile conditions, each PTFE membrane was cut into 10 mm circular discs and placed into cell culture plate wells each containing 900 μL Brucella broth medium (BD). A suspension of *C. albicans* cells (100 μL) from SDA medium, standardized to optical density (OD_600nm_) of 1, was added to each well except the yeast‐free control well. After 2 days of incubation, a small volume of the medium from each well was aliquoted to test for contamination. The membrane discs were transferred to the wells of a new sterile plate for washing with phosphate‐buffered saline to remove unbound and loosely bound *Candida* and the free DNA secreted by it in the medium. Subsequently, the membranes were transferred into sterile microcentrifuge tubes containing 150 μL nuclease‐free water, vortexed, and centrifuged at 10,000×*g* for 5 min to separate the intact *C. albicans* cells in the pellet. After discarding the supernatant, the pellet was then subjected to DNA purification.

### 2.4 Scanning electron microscopy (SEM)

Membrane discs with attached *C. albicans* from the above attachment assay were fixed in 3% glutaraldehyde in phosphate‐buffered saline (PBS) for 2 h on a rotator, followed by overnight refrigeration. The discs were then washed thrice in PBS, treated with 1% osmium tetroxide for 2 h, and dehydrated by sequential exposure to increasing concentrations of acetone (30%–100%) for 10 min each on a rotator. Subsequently, they were thoroughly dried in a critical point dryer, mounted on stubs with carbon double adhesive tape, coated with a layer of gold, and stored in a desiccator until examination under scanning electron microscope (JSM IT 200; JEOL).

### 2.5 DNA extraction and purification

DNA from both the reference *C. albicans* strain and the membrane‐detached *Candida* cells was purified using the DNeasy DNA Purification Kit (Qiagen GmbH) with an enzymatic lysis buffer comprising Tris ethylenediaminetetraacetic acid (EDTA) buffer (20 mM Tris, 2 mM EDTA), 1.2% Triton X‐100 and lysozyme. The resultant purified DNA was eluted in nuclease‐free water and quantified using UV spectrophotometry with NanoDrop^TM^ 1000 (Thermofisher).

### 2.6 Quantitative real‐time polymerase chain reaction (qPCR)

For qPCR, previously validated primers (Forward: TCA ACT TGT CAC ACC AGA TTA TT; Reverse: TCC TCC GCT TAT TGA TAT GC) targeting *C. albicans* 16S rRNA gene were used (Li et al., 2003). The qPCR reaction was performed as described earlier (Bhardwaj et al. 2020; PMID 32527216). Briefly, using a SYBR Green master mix (Power SYBR Green® Kit; Applied Biosystems), species‐specific primers (0.5 µL, nuclease‐free water (7 µL), and DNA template (2 µL) the reaction was run on ABI 7500 Fast RT‐PCR machine (Applied Biosystems). The data were analyzed using the associated SDS software 1.4.0v.

### 2.7 Statistical analysis

Each test was conducted in triplicates and yielded consistent results. Subsequently, the mean values were calculated and used for analysis. *C. albicans* quantities (cells) were log‐transformed after adding one to all data to handle zeroes in the statistical analyses. The test of significance was established by using nonparametric Mann–Whitney *U* test and a *p* value < .05 was considered significant.

## 3 RESULTS

### 3.1 SEM analysis of *C. albicans* attachment to membranes

**Figure 1:**
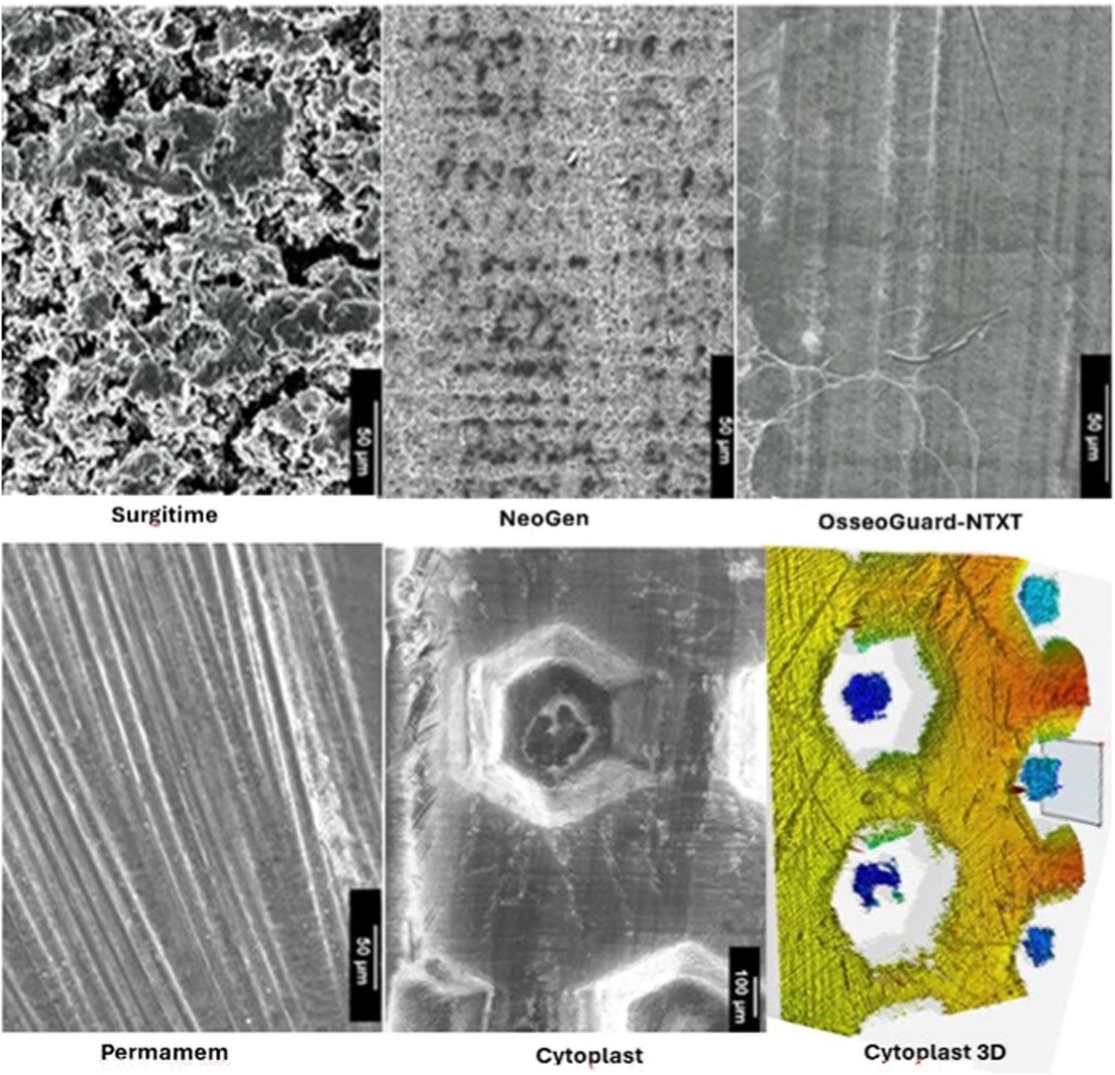
Scanning electron microscopy images of textured and nontextured polytetrafluoroethylene membranes showing surface roughness characteristics. The images are reproduced here after obtaining Copy Rights permission from the journal “materials” by the publisher MDPI (Qasim et al., 2023). NTXT, nontextured.

**Figure 2:**
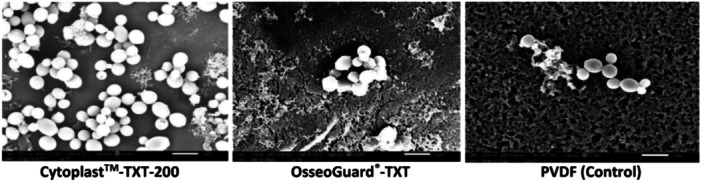
Scanning electron microscopy of *Candida albicans* attachment to textured and control polytetrafluoroethylene membranes. Bar = 1 µm, 10,000× magnification. PVDF, polyvinylidene fluoride; TXT, textured.

**Figure 3:**
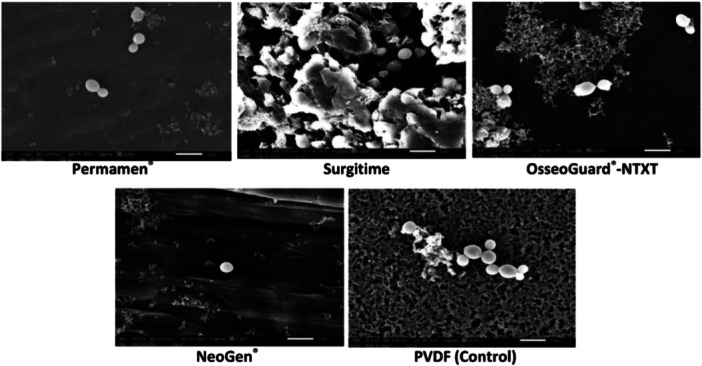
Scanning electron microscopy of *Candida albicans* attachment to nontextured and control polytetrafluoroethylene membranes. Bar = 1 µm, 10,000× magnification. PVDF, polyvinylidene fluoride; NTXT, nontextured.

The surface characteristics of the textured and nontextured PTFE membranes are visualized in the SEM images in Figure 1. *C. albicans* biofilms were grown on the PTFE membranes and subjected to SEM analysis. It was revealed that attachment of *C. albicans* cells to the PTFE membranes varied with the type of the membrane (textured and nontextured). The cells grew as microcolonies on the membranes with minimal attachment observed on the control membrane PVDF®‐P (Figures 2 and 3). Additionally, patchy and scattered attachment patterns were noted, along with small clusters of cells (Figures 2 and 3). The permamem® membrane showed primarily the cluster patterned attachment. Interestingly, on both the textured membranes (Cytoplast^TM^−200‐TXT and OsseoGuard®‐TXT; Figure 2) and the nontextured membranes (OsseoGuard®‐NTXT and Neogen®; Figure 3), only sparse attachment of tiny microbial clumps and free individual cells were observed without any biofilm. Surgitime was the sole nontextured membrane where *C. albicans* formed a thick biofilm (Figure 3). Notably, no difference in the microbial attachment was observed between OsseoGuard®‐TXT and OsseoGuard®‐NTXT (Figures 2 and 3).

### 3.2 Real‐time PCR quantification of *C. albicans* attached to the membranes

**Figure 4:**
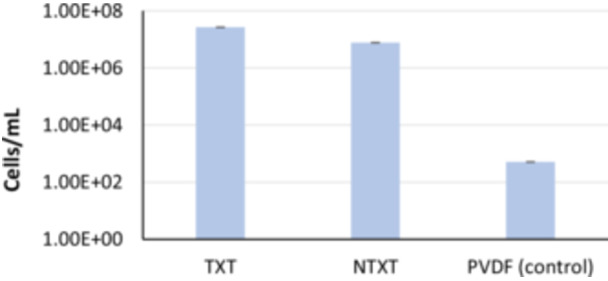
Quantification of *Candida albicans* attached to textured and nontextured polytetrafluoroethylene membranes by reverse transcription polymerase chain reaction. PVDF, polyvinylidene fluoride; NTXT, nontextured; TXT, textured.

The attachment of *C. albicans* to the PTFE membranes was assessed by analyzing the DNA purified from the membrane‐attached *C. albicans* and determining the number of cells attached to the membranes using quantitative RT‐PCR analysis. The results showed that the attachment to the PVDF® P membrane (0.0513 × 10^4^ cells/mL), which was used as a control membrane, was significantly poor compared to the textured (2680 × 10^4^ cells/mL) (*p* ≤ .001) and nontextured (707 × 10^4^ cells/mL) (*p* ≤ .001) PTFE membranes (Figure 4). Upon comparing the microbial attachment between the types of membranes, the *C. albicans* count was significantly higher in the group of textured membranes (2680 × 10^4^ cells/mL) than in the nontextured one (707 × 10^4^ cells/mL) (*p* = .001) (Figure 4).

**Figure 5:**
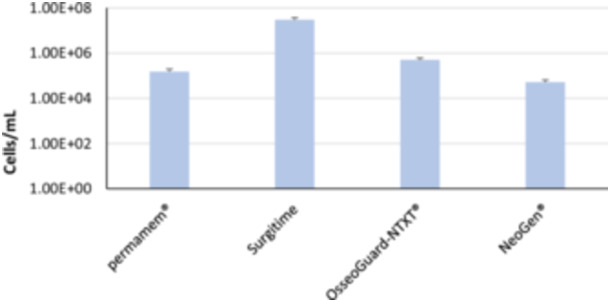
Quantification of *Candida albicans* attached to nontextured polytetrafluoroethylene membranes by reverse transcription polymerase chain reaction. NTXT, nontextured.

Among the nontextured membranes group (Figure 5), highest attachment of *C. albicans* was observed on Surgitime (3010 × 10^4^ cells/mL), which was significantly (*p* < .05) different from all other tested membranes.

## 4 DISCUSSION

In the present study, the attachment of *C. albicans* cells to six commercially available d‐PTFE membranes used for bone regeneration was investigated. SEM analysis revealed that *C. albicans* adhered to both textured and nontextured membranes in patchy, scattered, and small clustered patterns except for Surgitime with which it formed a thick biofilm. With quantitative analysis of attachment by RT‐PCR, we detected significantly greater number of *Candida* cells attached to PTFE membranes compared to the control membrane with textured membranes (2680 × 10^4^ cells/mL) exceeding the count than nontextured (707 × 10^4^ cells/mL) and Surgitime showing the highest count (3010 × 10^4^ cells/mL) among all examined membranes.

GBR procedures include the use of specifically designed surgical techniques aiming at maximally preserving the oral tissues followed by the application of various biomaterials which facilitate the regeneration of the bone defects. Because one of the main goals of periodontal therapy is to treat the infection caused by periodontal pathogenic biofilm it is reasonable to emphasize that regenerative surgery should only be considered after the completion of the infection control of the existing natural teeth and implants (Jepsen et al., 2023; Nibali et al., 2015; Sanz et al., 2020).

Oral microbial biofilms are three‐dimensional structured bacterial communities attached to a solid surface in the oral cavity, serving as an exemplary model system for bacterial attachment (Bos, 1999; Busscher & van der Mei, 1997). Not only numerous bacterial species participate in the development of biofilm complex structure but also fungi, especially *C. albicans*, that often commensally inhabits the oral cavity. *C. albicans* employs an extensive armory of various virulence factors supporting its coexistence with microorganisms resulting in successful host colonization and propagation of infection. Cross‐kingdom interactions between fungi and oral bacteria have drawn increasing attention.

The detection rate of *C. albicans* in the healthy population is 18.5%−40.9% (Babatzia et al., 2020; Du et al., 2022). *C. albicans* can interact with a variety of oral microbes and their interactions are interdependent and mutually beneficial rather than unidirectional. These polymicrobial interactions have been demonstrated in the pathogenesis of biofilm‐related oral diseases, including dental caries, oral candidiasis, endodontic diseases, periodontitis, implant‐related infections, and oral cancer. *C. albicans* is a symbiotic fungus commonly colonizing on the mucosal surfaces of living bodies (Du et al., 2022). Commensal bacteria increase not only the colonization of *C. albicans* in mucosal niches but also the persistence of *C. albicans*. The interaction between this fungus and oral bacteria may further modulate the virulence of *Candida* biofilm (Babatzia et al., 2020; Du et al., 2022). After adherence to the surface, *C. albicans* cells proliferate in the form of yeast and begin to form hyphal, elongating, and proliferating throughout the biofilm maturation process (Babatzia et al., 2020; Du et al., 2022; Mun et al., 2016). The yeast‐to‐hypha transition is widely recognized as a key virulence trait of *C. albicans* associated with biofilm formation. The synergistic effects of *C. albicans* and commensal bacteria have been well‐studied in the context of importance to the microbiological community, which have an impact on the virulence of polymicrobial biofilms and antibiotic resistance (Du et al., 2022; Morales & Hogan, 2010).

Fungal attachment on PTFE membranes has been barely studied; however, previous studies have demonstrated that *C. albicans* exhibit a strong affinity to adhere to surfaces made of high‐density polytetraethylene like PTFE (Grevstad & Leknes, 1993; Martins Leal Schrekker et al., 2023; Radford et al., 1999). It has been well established that the adherence of yeast and bacteria to such surfaces in the oral cavity and their accumulation as biofilms lead to increased inflammation of surrounding tissues (Naginyte et al., 2019). The findings of the current study suggest that the adherence of *C. albicans* on the exposed PTFE membrane in GBR can lead to the formation of a biofilm along with oral microorganisms and/or periodontal pathogens, which can have significant implications. It can lead to inflammation of the surrounded soft tissues (Naginyte et al., 2019), contribute to the development of oral candidiasis, and may also serve as a microbial reservoir for the recolonization and infection of the periodontal and/or peri‐implant area (Lafuente‐Ibanez de Mendoza et al., 2021; Souza et al., 2022). Previous in vivo studies have also supported this by demonstrating a significant presence of *C. albicans* adhering to and proliferating on implant surfaces and clinically isolating it from biofilms associated with peri‐implant diseases and implant failure (Arciola et al., 2018; Canullo et al., 2017; Lafuente‐Ibáñez de Mendoza et al., 2021).

The higher numbers of *C. albicans* detected on the surfaces of the textured membranes (Cytoplast^TM^−200‐TXT and OsseoGuard®‐TXT) in this study could be attributed to the roughness and the result of the existing hexagonal‐shaped indentations distributed evenly on these membranes' surface, as previously reported (Qasim et al., 2023). The purpose of these indentations is to enhance GBR stability by facilitating cell adhesion (Qasim et al., 2023); however, these are also susceptible to microbial contamination.

The higher numbers of *C. albicans* detected on the Surgitime surface compared to the other nontextured membranes could be favored by the high surface roughness of this material when compared to both textured and other nontextured membranes as demonstrated previously (Qasim et al., 2023). Furthermore, Surgitime and all other examined PTFE membranes were found to be hydrophobic as previously described (Qasim et al., 2023). There is enough evidence reported in a review by Quirynen et al. that rough surfaces promote cell adhesion and biofilm formation in the oral cavity (Quirynen & Bollen, 1995). Meanwhile, among various other factors, microbial cell attachment to surfaces depends strongly on the hydrophobicity of the microorganism (van Loosdrecht et al., 1990). A review by Radford et al. (1999) and the study of Minagi et al. (1985) strongly suggested that the hydrophobicity of *C. albicans* and its adherence to denture‐base materials are related (Radford et al., 1999). Furthermore, organisms with greater hydrophobicity adhere more strongly to hydrophobic surfaces, like PTFE membranes (Krasowska & Sigler, 2014). Hydrophobicity of *C. albicans* has been reported in several previous studies (Fukazawa & Kagaya, 1997; Hazen et al., 1990; Suchodolski et al., 2020). Furthermore, the hydrophobic surfaces have been shown to promote the formation of two phases in *C. albicans* biofilms. The first phase involves the adhesion of yeast cells to the surface forming a basal layer followed by a second phase involving the formation of the upper layer of hyphae embedded in extracellular polymeric matrix, which contributes to the structural integrity of the biofilm (Cavalheiro & Teixeira, 2018).

A major limitation of the present study may lie in the fact that only one *C. albicans* strain was used. More *C. albicans* strains, other candida species, and bacterial species need to be investigated in our future studies.

## 5 CONCLUSION

The results of this study indicate that *C. albicans* adhesion on membranes' surfaces depends on the degree of surface roughness and/or on the presence of a texture. Textured PTFE membranes and/or membranes' high roughness showed significantly more adhered *C. albicans* cells. These findings impact surgeon's choice of GBR membrane and postoperative maintenance.

## AUTHOR CONTRIBUTIONS

*Conceptualization and resources*: Gregor‐Georg Zafiropoulos. *Methodology*: Adel Al‐Asfour and Maribasappa Karched. *Analysis of the results and writing*: all authors. All authors have read and agreed to submit this manuscript for publication.

## CONFLICT OF INTEREST STATEMENT

The authors declare no conflict of interest.

## Data Availability

The processed data required to reproduce these findings cannot be shared at this time as the data also form part of an ongoing research.
